# Opto-CLIP reveals dynamic FMRP regulation of mRNAs upon CA1 neuronal activation

**DOI:** 10.1101/2024.08.13.607210

**Published:** 2024-08-14

**Authors:** Ruth A. Singer, Veronika Rajchin, Kwanghoon Park, Nathaniel Heintz, Robert B. Darnell

**Affiliations:** 1 Laboratory of Molecular Neuro-oncology, The Rockefeller University, New York, NY, USA; 2 Laboratory of Molecular Biology, The Rockefeller University, New York, NY, USA; 3 Howard Hughes Medical Institute, The Rockefeller University, New York, NY, USA

## Abstract

Neuronal diversity and function are intricately linked to the dynamic regulation of RNA metabolism, including splicing, localization, and translation. Electrophysiologic studies of synaptic plasticity, models for learning and memory, are disrupted in Fragile X Syndrome (FXS). FXS is characterized by the loss of FMRP, an RNA-binding protein (RBP) known to bind and suppress translation of specific neuronal RNAs. Since molecular studies have demonstrated that synaptic plasticity in CA1 excitatory hippocampal neurons is protein-synthesis dependent, together these observations have suggested a potential role for FMRP in synaptic plasticity in FXS. To explore this model, we developed a new experimental platform, Opto-CLIP, to integrate optogenetics with cell-type specific FMRP CLIP and RiboTag in CA1 hippocampal neurons, allowing investigation of FMRP-regulated dynamics after neuronal activation. We tracked changes in FMRP binding and ribosome-associated RNA profiles 30 minutes after neuronal activation. Our findings reveal a significant reduction in FMRP-RNA binding to transcripts encoding nuclear proteins, suggesting FMRP translational inhibition may be de-repressed to allow rapid translational responses required for neuronal homeostasis. In contrast, FMRP binding to transcripts encoding synaptic targets were generally stable after activation, but all categories of targets demonstrated variability in FMRP translational control. Opto-CLIP revealed differential regulation of subsets of transcripts within CA1 neurons rapidly after depolarization, and offers promise as a generally useful platform to uncover mechanisms of RBP-mediated RNA regulation in the context of synaptic plasticity.

## INTRODUCTION

The uniformity of DNA across different cell types underscores that the way in which genetic information is transcribed and processed underlies cellular diversity. In this view, careful examination of how RNA is spliced, polyadenylated, localized, and translated, will further our understanding of complex cellular functions. In neurons, RNA regulation has been of special interest to neuroscientists following the discovery that memory is protein synthesis dependent. The hippocampus is essential for new memory formation. Synaptic plasticity is the ability of synapses to strengthen or weaken over time in response to increases or decreases in their activity^1^. In hippocampal CA1 pyramidal neurons, synaptic plasticity can be demonstrated by electrophysiologic induction of long-term potentiation (LTP) and long-term depression (LTD). The induction of LTP in CA1 neurons is protein synthesis dependent and dendritically localized^2,3^.

Dysregulation of synaptic plasticity has been implicated in neurodevelopmental disorders, including Fragile X Syndrome (FXS)^4,5^. FXS, the most common inherited cause of intellectual disability and leading monogenic cause of autism, results from the silencing of the *Fmr1* gene and subsequent loss of its gene product, the RNA binding protein (RBP), FMRP^6,7^. Decades of research have uncovered the requirement of FMRP for proper neurophysiological function. *Fmr1*-KO mice exhibit defects in cognition and learning/memory formation^5,8–10^, dendritic spine morphology and dynamics^11^, and synaptic plasticity^12–14^, which shares similarities to human FXS phenotypes^15^. These studies formed the basis of a model whereby dysregulation of FMRP target RNAs leads to an imbalance of glutamate-dependent excitation/inhibition which underlies the development of intellectual disability in FXS^4,5,16^. Therefore, identifying the transcripts FMRP binds and regulates is fundamental to our understanding of synaptic plasticity and the pathophysiology of FXS^17^.

Direct FMRP targets have been identified using Crosslinking Immunoprecipitation (CLIP), in whole mouse brain^18,19^, CA1 excitatory hippocampal neurons^20^, and microdissected CA1 neurites and cell bodies^21^. CLIP revealed that FMRP binds across the coding sequence of mRNAs, and biochemical studies indicate that FMRP stalls ribosomes on these transcripts, indicating a direct role for FMRP in translational regulation^18,20–22^. Comparative analysis of FMRP binding and regulation revealed enrichment of transcripts related to Autism Spectrum Disorders^18,20^ and distinct regulation of synaptic and nuclear targets within the dendritic and cell body layers, respectively, of CA1 neurons^21^. These studies demonstrate how careful analysis of RBP-RNA interactions can lead to insight into mechanisms underlying intellectual disability in FXS. However, these analyses have been limited to steady state conditions, and therefore the precise mechanisms by FMRP globally regulates RNA in the context of synaptic plasticity remain incompletely understood.

Synaptic plasticity underlying learning and memory has historically been modeled physiologically^23,24^, behaviorally^25–27^, or chemically^28,29^ followed by a search for genes with modified expression. While these studies identified activity-induced transcriptional changes, such as immediate-early genes, the gene changes from different regions, times post activation, or stimulation paradigms had surprisingly little overlap^25,30^. The use of optogenetics to observe and control cellular activities has revolutionized the field of neuroscience^31,32^. Utilizing optogenetics to study synaptic plasticity has several advantages: manipulation is rapid, reversible, useful over short or long timescales, cell-type specific, and can be integrated with mouse models^31,33^. The rapid timescale of optogenetics is especially beneficial in the context of FMRP, which has been shown to be dephosphorylated in minutes following neuronal activation^13,34,35^. Optogenetics has the potential to uncover molecular mechanisms that underlie protein synthesis dependent synaptic plasticity.

Here, we describe the development and application of a new platform, termed Opto-CLIP, for the cell-type specific identification of RNAs regulated by FMRP in optogenetically activated excitatory CA1 hippocampal neurons. Our approach leverages Cre-dependent expression of channelrhodopsin (ChR2), GFP-tagged FMRP, and HA-tagged ribosomes specifically in CA1 neurons to study the molecular consequences of neuronal activation at the level of RNA-protein interactions and ribosome-associated transcripts. We observed significant changes in FMRP-binding patterns and ribosome-associated RNA profiles following optogenetic activation. By integrating data from Opto-FMRP-CLIP and Opto-RiboTag techniques, we have developed a comprehensive model of activity-induced FMRP-mediated RNA regulation. These results provide fresh insight into the dynamic regulation of RNA by FMRP in response to neuronal activation and provide a foundation for future research into the molecular basis of learning, memory, and neurodevelopmental disorders.

## RESULTS

### Optogenetic activation of excitatory CA1 neurons of the mouse hippocampus

To enable molecular analyses of FMRP-bound transcripts specifically in excitatory hippocampal neurons, we crossed Camk2a-Cre mice^36^ to either conditionally tagged (cTag)-FMRP mice^20,37^ or Rpl22-HA (RiboTag) mice^38^. This strategy enables cell-type specific expression of either GFP tagged FMRP (Fmr1-cTag) or HA tagged ribosomes (RiboTag). Previous studies found nearly identical CLIP results with GFP-tagged FMRP and endogenous FMRP, supporting the use of the Fmr1-cTag mouse for CLIP studies^37^. Furthermore, a similar strategy has been successfully implemented for other RNA-binding proteins, including NOVA2^39^ and PABPC1^40,41^.

**Figure 1.**
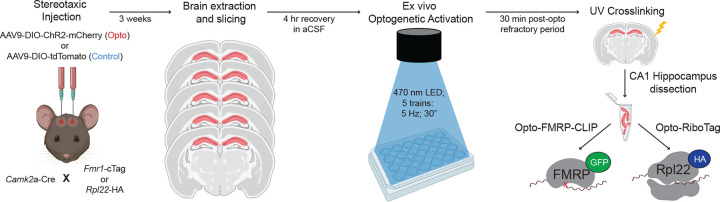
Overview of Opto-CLIP methodology Camk2a-Cre mice are crossed to either Fmr1-cTag mice or Rpl22-HA (RiboTag) mice. 8-week-old Camk2a-Cre;Fmr1-cTag (for FMRP-CLIP) or Camk2a-Cre;Rpl22-HA (for RiboTag) are stereotaxically injected with either AAV-DIO-ChR2-mCherry (Opto or Activated) or AAV-DIO-tdTomato (Control) to enable Cre-dependent expression of ChR2 (H134R) fused to mCherry or tdTomato with no opsin, respectively, in CA1 excitatory hippocampal neurons. 3-weeks post-injection, mice are sacrificed and acute brain slices are prepared. After 4 hour recovery in aCSF, all slices are exposed to an LED stimulus protocol (470 nm, 5 trains of 5 Hz; 30” each). Slices are then recovered for 30 minutes and UV crosslinked (CLIP). CA1 hippocampus is then dissected and samples are assayed by FMRP-CLIP or RiboTag.

**Figure 2.**
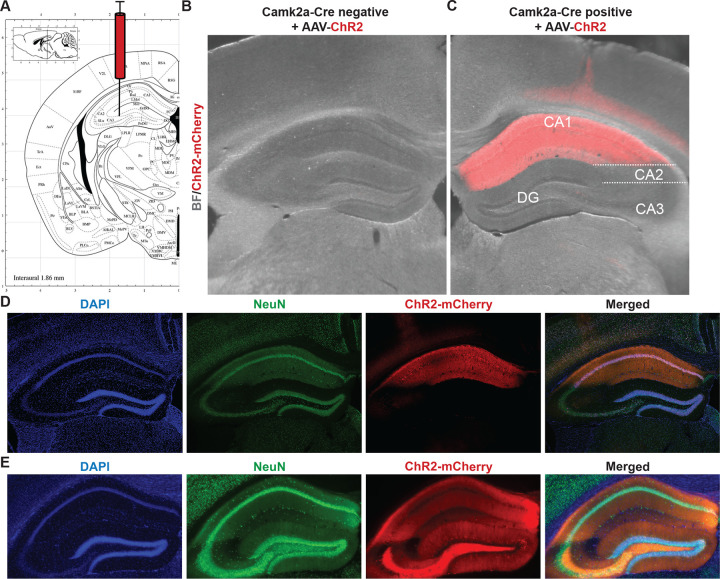
Cre-dependent expression of AAV-ChR2 in excitatory CA1 neurons of the adult mouse hippocampus. A) Schematic from Allen Brain Atlas showing region of the hippocampus targeted with AAVs. B and C) Overlaid Brightfield (gray) and fluorescent mCherry (red) images of the mouse hippocampus taken from Camk2a-Cre negative (B) and Camk2a-Cre positive (C) mice 3-weeks after stereotaxic injection of AAV-ChR2-mCherry. D) IF to detect AAV-mCherry expression in the mouse hippocampus 3-weeks after stereotaxic injection of AAV-mCherry. Injections took place at 8-weeks of age. DAPI (blue) marker for nuclei, AAV-ChR2-mCherry (red), and NeuN (green) to label all neurons. Magnification, 4X. E). IF to detect AAV-ChR2-mCherry expression in the mouse hippocampus 3-weeks after stereotaxic injection of AAV-mCherry. Injections took place at 12-weeks of age. DAPI (blue) marker for nuclei, AAV-mCherry (red), and NeuN (green) to label all neurons. Magnification, 4X.

In order to study the dynamics of FMRP-RNA regulation rapidly after CA1-specific depolarization, we developed Opto-CLIP (Figure 1) to allow for cell-type specific optogenetic activation of excitatory neurons in the mouse hippocampus. Opto-CLIP incorporates rapid UV-irradiation after activation, allowing identification of RNAs directly crosslinked to FMRP precisely at the time of irradiation. To optogenetically manipulate neurons prior to CLIP or RiboTag, we performed bilateral stereotaxic injections of a Cre-dependent adeno-associated virus (AAVs) to target the hippocampus (stereotaxic coordinates: M/L: +/− 2; D/V −2; A/P −2) (Figure 2A). ChR2 (Opto or activated) mice were injected with pAAV9-EF1a-DIO-hChR2(H134R)-mCherry, which expresses the channelrhodopsin-2 (ChR2)-mCherry fusion protein in a Cre-dependent manner. Control mice were injected with pAAV9-CAG-FLEX-tdTomato, which expresses the tdTomato reporter in all Cre expressing cells. Qualitative immunofluorescence (IF) confirmed that ChR2-mCherry expression was Cre-dependent (Figure 2B) and restricted to CA1 hippocampal neurons (Figures 2C and 2D). We empirically determined that waiting 3-weeks after AAV-injection achieved optimal expression of the ChR2 opsin (Figure 2D). Based on this timeframe, we sought to identify the ideal age of injection to restrict AAVs and molecular tags to area CA1 neurons. Consistent with previous reports^20,21,36^, we found that injecting mice at 8-weeks of age and sacrificing them for downstream molecular experiments at 11-weeks of age restricted Camk2a-Cre expression to CA1 neurons (Figures 2C and 2D). AAV injections at later ages (> 8-weeks) led to a more-widespread expression of Cre-reporters in CA3 neurons and the dentate gyrus (DG) (Figure 2E).

**Figure 3.**
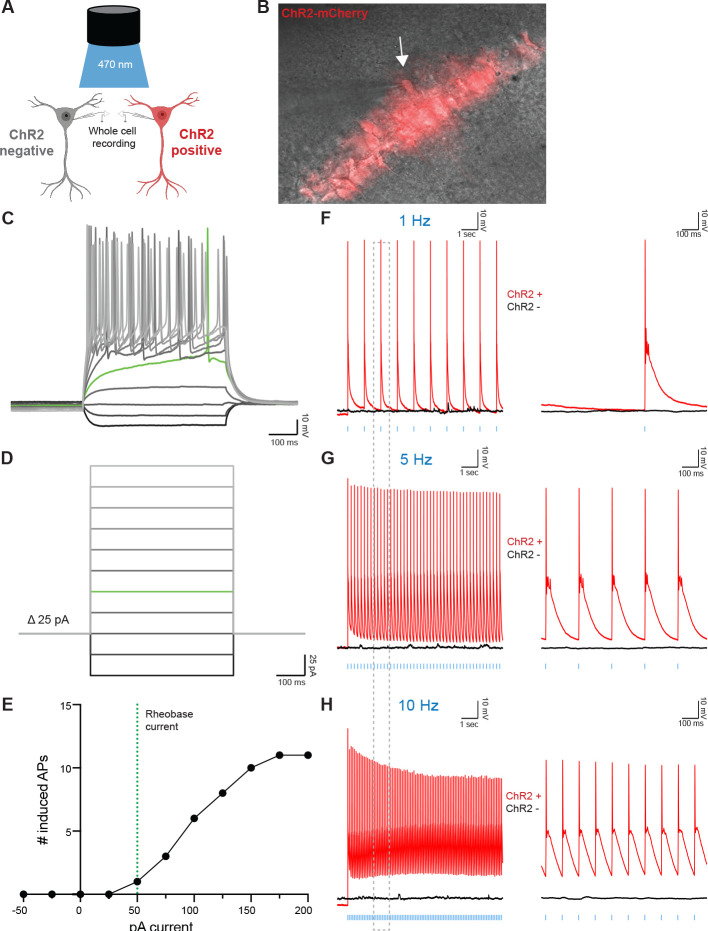
Optogenetic stimulation evokes action potentials in ChR2-expressing hippocampal excitatory neurons. A) Schematic of whole-cell configuration used for recordings of ChR2+ (visualized with red fluorescence) and ChR2- neurons B) Overlaid Brightfield (gray) and fluorescent mCherry (red) microscope image showing ChR2+ CA1 hippocampal neuron targeted for whole-cell patch clamping. Glass pipette tip and white arrow points to ChR2+ cell that underwent patch clamping. Magnification, 40X. C and D) Electrophysiological properties of ChR2+ neurons determined via stepwise injection of −50–200 pA current in 25 pA increments. Rheobase current, the minimal current to elicit an action potential (AP), was determined to be 50 pA (green lines) in ChR2+ neurons. Scale bars: 10 mV by 1 s. E) The number of AP induced by increasing injected currents showed that ChR2+ CA1 neurons had 50 pA rheobase current and fired multiple APs at low current injections. F-H) Whole-cell patch-clamp traces of ChR2+ (red lines) or ChR2- (black lines) neurons stimulated with 10 sec of blue LED light at 1 Hz (F), 5 Hz (G), or 10 Hz (H). Vertical blue dashes indicate a single LED pulse. Right panels are 1 second zoomed-in regions of left panels corresponding to dotted gray box. Scale bars are 10 mV by 100 ms.

In order to assess neuronal health following AAV injection and acute slice preparation, we sought to determine the intrinsic electrophysiological properties of CA1 pyramidal neurons. We injected Camk2a-Cre mice with AAV-ChR2-mCherry and performed fluorescence-guided whole-cell current clamp recordings in ChR2 positive neurons (Figures 3A and 3B). We observed voltage decreases in response to a −50 pA and −25 pA hyperpolarizing current injections (Figures 3C and 3D). Rheobase current, the minimal current to elicit an action potential (AP), was deter mined to be 50 pA in ChR2 positive CA1 neurons (Figures 3C–3E). Depolarizing current injections greater than 50 pA resulted in repetitive burst firing (Figures 3C–3E). AP frequency-current curves generated by the stepwise current injections revealed that ChR2 positive neurons increase AP firing in response to current injections and plateau at sustained AP frequencies upon 175 pA current injection (Figure 3E). In order to determine optimal optogenetic activation parameters for molecular analyses, we performed whole cell recordings on ChR2 positive and ChR2 negative neurons during exposure to blue light. ChR2 positive, but not ChR2 negative, neurons displayed time-locked AP firing in response to blue light pulses at 1 Hz (Figure 3F), 5 Hz (Figure 3G), and 10 Hz (Figure 3H) frequencies. All three frequencies successfully induced a single APs per light pulse for the entire duration of the stimulation protocols (10 seconds). Taken together, these data demonstrate that Cre-dependent ChR2 expression in CA1 neurons allows for optogenetic manipulation and establishes a paradigm to study cell-type specific RNA regulation following neuronal activation.

### Opto-CLIP identifies FMRP targets in control and activated neurons

A key innovation of using optogenetics as an activation paradigm to study FMRP-mediated RNA regulation is that both optogenetic activation and molecular analyses are done in a cell-type specific manner. Specifically, Camk2a-Cre activity drives expression of ChR2 and HA-tagged ribosomes or GFP-tagged FMRP; the same neurons that are optogenetically activated can be molecularly assayed without the need for physical isolation, which could alter the RBP-RNA interactions.

**Figure 4.**
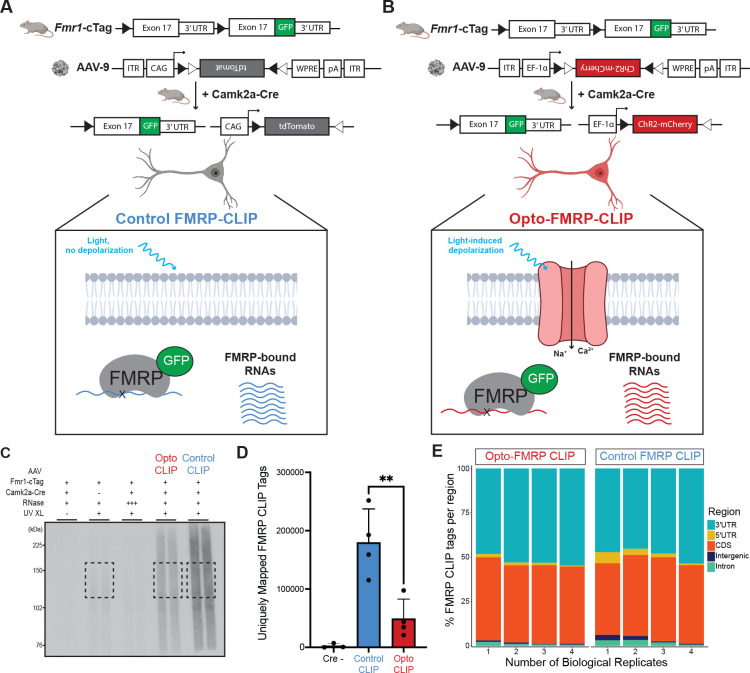
Opto-FMRP-CLIP uncovers RNAs differentially bound by FMRP in optogenetically activated neurons. A and B) Schematic depicting genetic targeting strategy for Control FMRP-CLIP (A) and Opto-FMRP-CLIP (B). The final exon of the Fmr1 gene is flanked by loxP sites and a second copy of this exon with an additional AcGFP sequence at the end of the coding region is cloned downstream. Cre-dependent recombination causes usage of the alternative final exon and expression of the GFP-tagged FMRP. Crossing Fmr1-cTag mice with Camk2a-Cre mice enables cell-type specific expression of GFP-tagged FMRP in excitatory neurons of the hippocampus. 3-weeks after stereotaxic injection of AAV-Control (A) or AAV-ChR2 (B), acute brain slices are exposed to LED activation paradigms which induces cell-type specific depolarization followed by UV crosslinking and molecular analysis by FMRP-CLIP. C) Autoradiograph image from Control and Opto-CLIP. Negative control samples consist of no UV Crosslinking (XL) (lane 1), Cre negative (lane 2), and high RNase treatment (lane 3). Black boxes indicate regions (120–175 kDa) subjected to CLIP sequencing D) Comparison of uniquely mapped FMRP-CLIP tags obtained in replicate Control and Opto-CLIP experiments. See Methods for details of computational methods used to obtain uniquely mapped tags. Significance was calculated using a two-tailed, unpaired Student’s t-test. ** <0.01. n=3, Cre negative samples. n=4 for Control and Opto-CLIP samples. E) Genomic annotations of mapped CLIP tags separated by biological complexity (BC). See also Figure S1.

To define FMRP-binding maps in optogenetically activated CA1 neurons, we modified the standard electrophysiology protocol (Figure 3) to enable parallel processing of large sample sizes required for molecular assays. Camk2a-Cre;Fmr1-cTag mice were injected with either AAV-Control (Figure 4A) or AAV-ChR2 (Figure 4B) and 3 weeks later acute brain slices were prepared and allowed to recover for four hours to promote the return to baseline gene expression levels. Slices were then exposed to an LED stimulation protocol, recovered for 30 minutes, and then UV-crosslinked. To enrich for CA1 neurons and deplete GFP-expressing neurons from CA3 and DG, area CA1 was dissected prior to CLIP.

To isolate FMRP-bound RNA, crosslinked samples were immunoprecipitated with anti-GFP antibodies, and RNA fragments were isolated, stringently purified, and sequenced (Figure 4C). Three cohorts of Cre negative samples were used as a negative control and processed in parallel (Figure 4C, second lane). Reads were mapped to the transcriptome and the number of uniquely mapped tags (UMTs) was determined for each replicate. Cre negative samples had very few UMTs compared to Cre positive samples (Figure 4D), demonstrating the low signal to noise of FMRP-CLIP. A ~3.5-fold decrease in FMRP binding was seen in light-treated neurons compared to controls (Figure 4D). Principal component analysis (PCA) revealed that Opto-CLIP and Control replicates clustered as distinct groups (Figure S1A) and had high R^2^ values (Pearson correlation, R^2^ > 0.82 for Opto-CLIP replicates; R^2^ > 0.92 for Control replicates) when comparing mapped tags per transcript across samples (Figure S1B). These results demonstrate that Opto-CLIP yielded reproducible results across biological replicates. Mapping the genomic distribution of tags with increasing biological complexity (requiring a tag to be found in “n” biological replicates) revealed a preference for CDS and 3’ UTRs, with Opto-CLIP and Control samples having similar distributions (Figure 4E), consistent with prior observations of FMRP CLIP in mouse brain^18,20,21^. In total, 3661 genes contained at least 5 FMRP CLIP tags in all four Opto-CLIP or Control experiments (BC =4). Taken together, Opto-FMRP-CLIP accurately defined RNAs bound by FMRP in activated neurons.

### Opto-RiboTag identifies ribosome-associated transcripts in control and activated neurons

For several well-studied RBPs, quantification of differential binding between two conditions has been accomplished by pooling CLIP tags from multiple replicates, calling peaks, and then qualitatively comparing tags in peaks across conditions^40,42,43^. FMRP, however, has been shown to display continuous coverage across the coding sequence of its targets^18,20,21^, and consequently traditional peak-calling algorithms fail to capture the nature of FMRP binding to its target transcripts. As an alternative, studies have mapped FMRP CLIP tags to the transcriptome and normalized FMRP binding relative to transcript abundance in that same cell type^20,21^. To measure transcript abundance, we crossed Camk2a-Cre mice with the RiboTag mouse^38^, enabling Cre-mediated HA tagging of all ribosome bound transcripts in CA1 neurons. Immunoprecipitation (IP) with an HA antibody, in the presence of cycloheximide to maintain ribosome-mRNA association, enables isolation of all ribosome-bound transcripts. Although this strategy limits quantitation to ribosome-bound RNAs, FMRP is predominantly associated with polyribosomes in the brain^18^, and in-depth analyses have demonstrated cell-type specific RiboTag efficiently isolates the majority of the transcriptome^20,21,44–46^.

**Figure 5.**
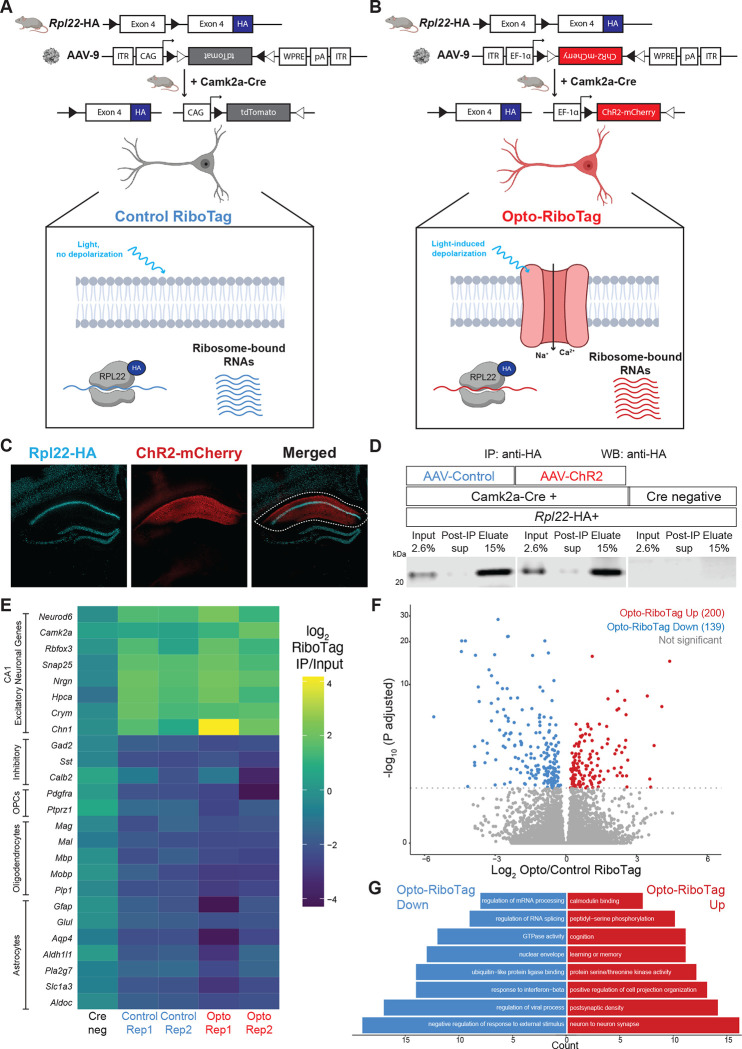
Opto-RiboTag robustly defines ribosome-bound RNA in optogenetically activated and control neurons. A and B) Schematic depicting genetic targeting strategy for Control RiboTag (A) and Opto RiboTag (B). Crossing Rpl22-HA mice with Camk2a-Cre mice enables cell-type specific expression of HA-tagged ribosomes in excitatory neurons of the hippocampus. 3-weeks after stereotaxic injection of AAV-Control (A) or AAV-ChR2 (B), acute brain slices are exposed to LED activation paradigms which induces cell-type specific depolarization and molecular analysis is performed by HA-IP and RNA-sequencing of input and immunoprecipitated RNA. C) Microscope images showing IF done on brain slices from Camk2a-Cre;Rpl22-HA mice 3-weeks following stereotaxic injection of AAV-mCherry. DAPI (blue) marker for nuclei, AAV-mCherry (red), and HA (cyan) to label Camk2a-Cre responsive neurons that will undergo optogenetic activation and molecular analysis by RiboTag neurons. White dotted line indicates the CA1 hippocampus that is dissected prior to RiboTag. Magnification, 10X. D) Western blot for HA shows that HA protein is restricted to Camk2a-Cre+ samples (left 6 lanes), is sufficiently depleted from cell lysate by immunoprecipitation (IP) with HA-antibody (Post-IP sup lanes), and is enriched in eluate following HA-IP. Marker showing size of 20 kDa ladder. E) Heatmap of the RiboTag enrichment scores calculated by log2 (RiboTag IP Transcript Per Million (TPM)/RiboTag InputTPM) following HA-immunoprecipitation from Cre negative and Cre positive RiboTag samples. Yellow color indicates positive enrichment of CA1 excitatory neuronal genes and dark blue color indicates negative enrichment of non-excitatory markers, such as inhibitory, OPC (oligodendrocyte precursor cells), oligodendrocytes, and astrocytes genes. Cre negative sample is from Cre negative;Rpl22-HA mice subjected to the same RiboTag pipeline as the other samples. F) Volcano plots showing RNA transcripts upregulated (red) and downregulated (blue) in Opto RiboTag versus Control RiboTag. Transcripts are considered significant with a p.adjusted < 0.05. Significance was calculated from DESeq2 with correction for multiple testing correction done via the Benjamini–Hochberg method. G) Gene Ontology (GO) analysis on ribosome bound RNAs downregulated (blue/left) or upregulated (red/right) following optogenetic activation. Counts indicate the number of genes found in each category. All categories listed are p.adjusted (Benjamini–Hochberg) < 0.05 measured by enrichGO^58^.

To define ribosome associated transcripts in activated CA1 neurons, we injected Camk2a-Cre;Rpl22-HA mice with either AAV-Control (Figure 5A) or AAV-ChR2 (Figure 5B) and performed Control and Opto-RiboTag. Brain slices were optically activated and dissected (Figure 5C, white dotted region) as for Opto-CLIP, excluding the crosslinking step. Rpl22-HA positive, Camk2a-Cre negative mice were used as controls and processed in parallel (Figure 5D; right three lanes; Figure 5E; left column). We optimized the concentration of HA antibody used to maximize HA-depletion post-IP (Figure 5D). We detected similar levels of HA protein in ChR2 and Control samples and HA protein was absent in Cre negative samples (Figure 5D). Cre positive samples, but not Cre negative samples, had enrichment (log2 FC IP/input > 0) of CA1 specific excitatory neuronal genes (*Neurod6, Camk2a, Rbfox3, Snap25, Nrgn, Hpca, Crym,* and *Chn1*), while markers of other cell types, such as inhibitory neurons (*Gad2, Sst*, and *Calb2*), oligodendrocyte precursor cells (OPCs) (*Pdgfra* and *Ptprz1*), oligodendrocytes (*Mag, Mal, Mpb, Mobp,* and *Plp1*), and astrocytes (*Gfap, Glul, Aqp4, Aldh1l1, Pla2g7, Slc1a3,* and *Aldoc*) were de-enriched (log2 FC IP/input < 0) (Figure 5E). These data demonstrate that mRNAs isolated by RiboTag IP originate from CA1 excitatory neurons.

Sequencing analyses of Control and Opto-RiboTag samples uncovered RNAs differentially bound by ribosomes upon activation, of which 139 transcripts were upregulated and 200 transcripts were downregulated (Figure 5F; FDR < 0.05). To explore pathways involved in molecular changes following optogenetic activation, we performed gene set enrichment analysis (GSEA) on upregulated and downregulated transcripts from Control and Opto-RiboTag. Interestingly, gene sets associated with learning and memory, cognition, and postsynaptic density were enriched in transcripts upregulated in Opto-RiboTag (Figure 5G). In contrast, downregulated transcripts encode proteins involved in RNA splicing, mRNA processing, GTPase activity, and negative regulation of response to external stimulus (Figure 5G). This indicates that different biologically coherent sets of ribosome transcripts–those involved in synaptic plasticity and higher order levels of RNA regulation, respectively–are preferentially ribosome bound after depolarization. Taken together, these data demonstrate that Opto-RiboTag is a sensitive technique for uncovering cell-type specific changes in RNA regulation following neuronal activation.

### FMRP targets are dynamically regulated in activated neurons

**Figure 6.**
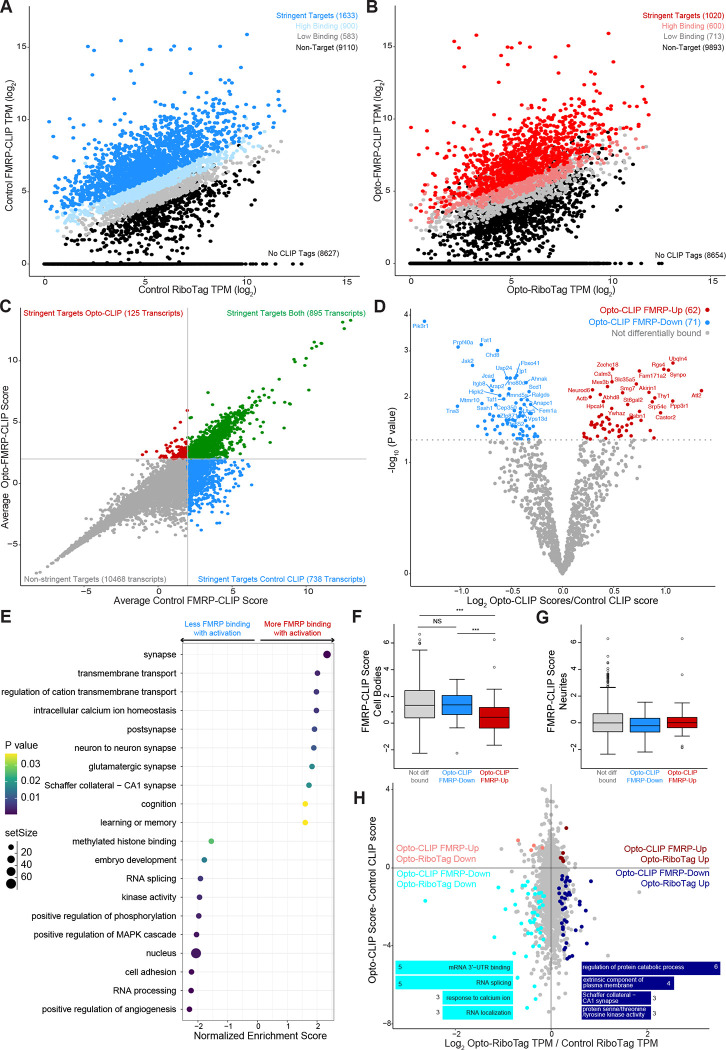
FMRP alters binding in activated neurons which triggers dynamic regulation of transcripts essential for neuronal function. A and B) Scatter plots of log2 normalized transcript per million (TPM) of CLIP tags from averaged Control (A) or Opto-CLIP (B) experiments compared to average log2 RiboTag TPM from Control (A) or Opto-RiboTag (B). n=4 biological replicates for FMRP-CLIP. n=2 biological replicates for RiboTag. Stringent targets were defined as those with an average CLIP score > 2, targets with high binding were defined as those with an average CLIP score > 1 and targets with low binding were defined as those with an average CLIP score between 0 and 1. Targets of each subclass are highlighted in the plot and the number of genes within each subclass is indicated. See methods for further details on CLIP score calculations. C) Scatter plot of Opto-CLIP scores compared to Control CLIP scores for each transcript. Green dots indicate transcripts with CLIP scores > 2 in both conditions. Blue dots indicate transcripts with CLIP scores > 2 in control neurons and < 2 in activated neurons. Red dots indicate transcripts with CLIP scores < 2 in control neurons and > 2 in activated neurons. Gray dots indicate transcripts with CLIP scores < 2 in both conditions. D) Volcano plot comparing log2 fold changes and p-values comparing Control versus Opto-CLIP scores (significance determined by Limma^57^, p-value < 0.05). Transcripts with significantly higher Opto-CLIP scores are shown in red (FMRP-Up). Transcripts with significantly lower Opto-CLIP scores are shown in blue (FMRP-Down). E) Gene Set Enrichment Analysis (GSEA) on transcripts differentially bound by FMRP as shown in D. Positive normalized enrichment score (NES) indicates transcripts more bound by FMRP in activated conditions (red dots in D) and negative NES indicates transcripts less bound by FMRP in activated conditions (blue dots in D). F and G) Boxplots showing FMRP CLIP scores calculated from microdissected CA1 cell bodies (F) and neurites (G)^21^. Red boxes show transcripts with significantly higher Opto-CLIP scores. Blue boxes show transcripts with significantly lower Opto-CLIP scores. Wilcoxon rank-sum test was used to determine significance (*** = p-value < 0.0001; NS = p-value > 0.05). H) Scatter plot of Opto-RiboTag versus Control RiboTag (log2 TPM) compared to differential CLIP scores (Opto-CLIP Score - Control CLIP Score). Gray dots indicate transcripts that are stringently bound by FMRP but are not significantly changed in Ribo Tag. Salmon dots indicate transcripts that are more bound by FMRP in activated neurons (FMRP-Up) and are downregulated in Opto-RiboTag. Dark Red dots indicate transcripts that are more bound by FMRP in activated neurons (FMRP-Up) and are upregulated in activated RiboTag. Cyan dots indicate transcripts that are less bound by FMRP in activated neurons (FMRP-Down) and are downregulated in activated RiboTag. Dark blue dots indicate transcripts that are less bound by FMRP in activated neurons (FMRP-Down) and are upregulated in activated RiboTag. Inset of graph: GO analysis on transcripts differentially regulated by FMRP-CLIP and differentially expressed by RiboTag. Cyan bars correspond to cyan dots. Dark blue bars correspond to dark blue dots. Bar length corresponds to the number of genes mapping to a GO category. All categories listed are p.adjusted (Benjamini–Hochberg) < 0.05 measured by enrichGO^58^. See also Figure S2.

We next sought to integrate our findings from Opto-RiboTag and Opto-FMRP-CLIP to build a model of FMRP-mediated RNA regulation in activated neurons. Previous studies demonstrated that FMRP CLIP tag density on a specific transcript is significantly correlated with the abundance of the transcript^20,21,46^; however, analyses was limited to steady state conditions. To determine if this finding holds true in activated neurons, we compared transcript abundance in Opto-RiboTag and Opto-FMRP-CLIP samples. Parallel analyses were done to compare Control RiboTag to Control FMRP-CLIP. We found that transcript abundance measured by RiboTag and CLIP were significantly positively correlated in both control and activated conditions (Figures S2A and S2B; Spearman correlation test; p-value < 2.2e-16 in all 8 samples). We used this relationship to calculate an FMRP CLIP score for each transcript that quantifies the amount of FMRP binding to a transcript relative to other transcripts of similar abundance. We compared average Control or Opto-RiboTag (log_2_ TPM) to each individual Control or Opto-CLIP (log_2_ TPM) replicate and fit each plot with a linear regression line (Figures S2A and S2B). For each transcript, we calculated Control and Opto-CLIP scores so that the higher the score, the greater the FMRP binding. For each condition, FMRP binding stringency was calculated by averaging Control and Opto-CLIP scores across replicates. In control neurons, there were 1633 stringent FMRP targets (Figure 6A) compared to 1020 stringent targets in activated neurons (Figure 6B). To determine how FMRP binding changed with optogenetic activation, we further classified all transcripts based on Control and/or Opto-FMRP-CLIP scores. 895 transcripts had high CLIP scores in both activated and control conditions (Figure 6C, green dots), 125 transcripts had only high Opto-FMRP-CLIP scores (Figure 6C, red dots), 738 transcripts had high Control CLIP scores (Figure 6C, blue dots), and most (10468) transcripts had neither (Figure 6C, gray dots). These data identify stringent FMRP targets in control and activated neurons.

To quantify the change in FMRP binding after activation, we compared CLIP scores in control versus activated neurons for each transcript. We discovered 62 transcripts had significantly higher CLIP scores in activated neurons compared to control neurons (Figure 6D, red dots, herein referred to as “FMRP-Up”), while 71 transcripts had significantly lower CLIP scores in activated versus control neurons (Figure 6D, blue dots, herein referred to as “FMRP-Down”). Interestingly, when examined by GSEA analysis, “FMRP-Up” transcripts were enriched in synaptic functions, such as transmembrane transport, glutamatergic synapses, learning and memory, cognition, and intracellular calcium ion homeostasis (Figure 6E). In contrast, “FMRP-down” transcripts were associated with nuclear functions, such as RNA processing, RNA splicing, and methylated histone binding (Figure 6E). These findings were reminiscent of prior data indicating that FMRP may have distinct RNA binding preferences in subcellular regions of CA1 neurons^21^. Hence, we compared FMRP CLIP scores in transcripts previously identified as FMRP cell body targets. Remarkably, we found that transcripts encoding synaptic functions (FMRP-Up) had significantly lower cell body CLIP scores than “FMRP-Down” transcripts associated with nuclear functions (Figure 6F). This observation was in agreement with our GSEA analysis (Figure 6E). We did not observe any significant differences in FMRP CLIP scores previously identified as FMRP dendritic targets after neuronal activation (Figure 6G). These findings demonstrate that after activation of CA1 neurons, FMRP is less bound to transcripts involved in nuclear RNA regulatory processes. FMRP binding to synaptic transcripts showed no such change (Figure 6G). Taken together, this observation suggests the possibility that there is differential regulation of FMRP binding in the cell body subcellular compartment after depolarization, and is discussed below.

Our observation that FMRP binds fewer overall transcripts in activated neurons (1633 transcripts in Control CLIP (Figure 6A) versus 1020 transcripts in Opto-CLIP (Figure 6B)) led us to hypothesize that FMRP may be released from ribosome binding on some transcripts upon neuronal activation. Given previous work showing FMRP acts as a reversible repressor of translation^18,20,21^, we overlaid differential FMRP-CLIP binding and differential transcript abundance measured by RiboTag (Figure 6H). Of the transcripts that were less bound by FMRP in activated neurons (FMRP-Down), 42 transcripts had decreased ribosome binding after activation (Figure 6H, cyan dots) and 38 transcripts had increased ribosome binding after activation (Figure 6H, dark blue dots). These data demonstrate that changes in FMRP binding can both positively and negatively influence transcript abundance, as measured by RiboTag, and suggests that within the cell body, FMRP-regulated transcripts may be “translationally de-repressed” (as evidenced by less FMRP binding and increased ribosome binding).

To investigate potential functional differences between transcripts differentially bound by Opto-FMRP and differentially expressed by Opto-RiboTag, we performed GSEA analysis on translationally de-repressed transcripts identified above (Figure 6H, colored dots). We found that transcripts less ribosome-associated and less bound by FMRP in activated neurons were significantly enriched in pathways involving 3’-UTR binding, RNA splicing, response to calcium ion, and RNA localization (Figure 6H, cyan bars). In contrast, transcripts that were more ribosome-associated and less bound by FMRP in activated neurons were significantly enriched in genes involved in regulation of protein catabolic process, extrinsic component of plasma membrane, Schaffer collateral-CA1 synapse, and protein serine/threonine/tyrosine kinase activity (Figure 6H, dark blue bars). Taken together, neuronal activation induces changes in FMRP binding causing up and down regulation of functionally distinct classes of mRNAs.

## DISCUSSION

FMRP is required for key neuronal functions, such as synaptic plasticity underlying learning and memory^4,5,12,13,16^, yet global FMRP:RNA interactions during neuronal activation have not been defined. Here we describe a new platform, termed Opto-CLIP, to investigate cell-type-specific RNA regulation by FMRP-CLIP in optogenetically activated CA1 excitatory neurons. Opto-CLIP revealed significant changes in FMRP-binding patterns and ribosome-associated RNA profiles upon neuronal activation.

We find that FMRP stringently binds two distinct subsets of FMRP targets. One set of transcripts showed increased FMRP binding after optogenetic activation (Opto-CLIP FMRP-Up). These transcripts were enriched for genes controlling synaptic functions. The simplest interpretation here is that FMRP binds these transcripts to block ribosomal elongation^18^ and acutely downregulate their further translation after depolarization. This is consistent with the observation that at least some of (6/125) of these transcripts show increased ribosomal binding (increased RiboTag) after activation (Figure 6H). Moreover, given that the synaptic FMRP targets have been defined in CA1 neurons to be enriched in dendrites versus CA1 cell bodies, our data would suggest a rapid and local action of FMRP to inhibit translation of synaptic targets after activation.

Conversely, we find a different set of transcripts that show decreased FMRP binding after activation (Figure 6D), and remarkably, these transcripts encode a different set of proteins, those involved in nuclear processes, such as RNA splicing and histone binding. This suggests that FMRP translational repression may be relieved for processes involved in nuclear functions, and is particularly intriguing since FMRP has previously been shown to bind to such transcripts preferentially in the CA1 cell soma^21^.

Together, the differing FMRP actions on these two different biologic groups of targets is consistent with a model in which FMRP acts differentially in different subcellular compartments of the same CA1 neuron. In the first—the synaptic dendrite, there is a quick feedback to inhibit translation and consequent synaptic signaling, and the second—the cell soma, there is a release of translational inhibition and generation of proteins involved in nuclear regulation. In this model, FMRP sits as a regulatory sensor of activation, triggering different and biologically coherent responses appropriate to different subcellular compartments. More generally, the data encourage further exploration of a model relating FMRP to dampening synaptic signaling and increasing transcriptional/nuclear RNA regulatory controls after depolarization. Evidence supporting such a model would place FMRP as an integrator linking activity from the synapse to the nucleus to regulate homeostatic plasticity^17,47^.

Of the RNAs less bound by FMRP with activation, roughly equivalent numbers were upregulated and downregulated by RiboTag. This suggests that FMRP can up or downregulate translation of some transcripts. This is supported by previous work that identified transcripts both translationally up and downregulated by FMRP^48^, including those identified by TRAP in *Fmr1*-KO versus WT CA1 hippocampus^49^. Another consideration is that FMRP can act in different time courses on different transcripts. In this study, we examined binding at 30 minutes after depolarization based on previous work examining multiple layers of RNA regulation in activated neurons^50^. It is plausible that FMRP has rapid and slower translational controls within transcripts and/or cell compartments. Future experiments investigating the temporal dynamics of FMRP-mediated RNA regulation will provide key insights into these unknown.

While our study introduces significant advancements in the understanding of FMRP-mediated RNA regulation, there are caveats to consider. Optogenetic activation paradigm, though advantageous for its rapid and reversible nature, may not fully recapitulate the complexities of in vivo neuronal activation and synaptic plasticity. Additionally, while we demonstrated significant changes in FMRP binding and ribosome association upon neuronal activation, the functional implications of these changes remain to be fully elucidated. Future studies incorporating electrophysiology and protein analysis will be important. Expanding this approach to include other neuronal subtypes and brain regions will provide a more comprehensive understanding of FMRP’s role across the nervous system. We note that specific transcripts and pathways that are differentially regulated by FMRP upon neuronal activation could serve as potential therapeutic targets for modulating synaptic function and treating cognitive impairments associated with FXS and other neurodevelopmental disorders. Moreover, Opto-CLIP should be widely applicable for studies on other RNA binding proteins, and integration of these datasets will be crucial for furthering our understanding of the role of RBP-mediated RNA regulation in neuronal function and dysfunction.

In conclusion, this study introduces a powerful platform for studying cell-type-specific RNA regulation by RNA binding proteins in response to neuronal activity. Our integrated approach combining Opto-FMRP-CLIP and Opto-RiboTag enabled the construction of a comprehensive model of FMRP-mediated RNA regulation. These insights shed light on the molecular mechanisms underlying synaptic plasticity and cognitive functions and lay the foundation for future research into the molecular basis of learning, memory, and neurodevelopmental disorders.

## MATERIALS AND METHODS

### Mice

All animal procedures were approved by The Rockefeller University Institutional Animal Care and Use Committee (IACUC) and mice were treated in accordance with the National Institutes of Health Guide for the Care and Use of Laboratory Animals. Mice were allowed ad libitum access to food and water at all times, weaned at 3 weeks of age, and maintained on a 12-hour light/dark cycle. All mouse lines used in these studies were on the C57BL/6 genetic background. B6.Cg-Tg (Camk2a-cre)T29–1Stl/J (Camk2a-Cre)^36^, B6N.129-Rpl22tm1.1Psam/J (RiboTag)^38^ were purchased from Jackson Laboratories. The Fmr1-cTag mouse has been described and characterized elsewhere^37^. Briefly this mouse line was generated by introducing loxP sites either side of the terminal exon of the Fmr1 gene followed by a downstream AcGFP-tagged version of the terminal exon and surrounding intronic sequences. Thus, either FMRP or AcGFP-tagged FMRP can be expressed from the cTag allele in a mutually exclusive manner, dependent on Cre expression.

### Stereotaxic Surgeries

Stereotaxic surgeries were performed on 8-week-old Camk2a-Cre mice crossed to either Fmr1-cTag mice or Rpl22-HA mice. Mice were anesthetized with inhaled isoflurane (Kent Scientific; SomnoFlo) at 3% for induction. Once anesthetized, animals were placed in the stereotaxic frame (Kopf; Model 1900) and exposed to 0.25% isoflurane for the remainder of surgery. Mice were shaved and paralube (artificial tears) applied to the eyes to avoid drying. The surgical field was cleaned with betadine solution followed by 70% ethanol. A sterile scalpel was used to perform a midline incision to expose the skull. The position of bregma was determined and used as a landmark for stereotaxic coordinates. The following coordinates (in mm) were used for targeting CA1 neurons (in mm: Anterior/Posterior: −2, Medial/Lateral: +/−2, Dorsal/Ventral: −2). Two small burr holes (0.6 mm) were drilled bilaterally over the target site of injection. A 34G Beveled needle (World Precision Instruments; NF34BV-2) attached to a 10 μl Hamilton syringe was used to inject one microliter of virus per site at a rate of 0.095 μl/min. Following virus injections, the needle was left in place for 5 minutes before being slowly retracted. For conditional expression of channelrhodopsin in the mouse hippocampus, mice were injected with pAAV-EF1a-double floxed-hChR2(H134R)-mCherry-WPRE-HGHpA (a gift from Karl Deisseroth; Addgene: 20297). For conditional expression of the control reporter, mice were injected with pAAV-FLEX-tdTomato (a gift from Edward Boyden; Addgene: 28306). Both plasmids were AAV9 serotypes and were diluted 1:20 which corresponded to 1.25 E+09 genomic particles per injection site. The scalp incision was closed with dissolvable surgical sutures (4–0 Coated Vicryl Violet 1×27” FS-2; Ethicon; J397H) and mice were given a subcutaneous injection of Meloxicam SR (6 mg/kg) for pain relief that lasts 48 hours. In addition, 1.0 mL sterile saline was administered subcutaneously to prevent further dehydration. Mice were allowed to recover from surgery for 3 weeks prior to any downstream experiments.

### Brain Slice Preparation

At 11 weeks of age, mice were deeply anesthetized with 3% inhaled isoflurane (Kent Scientific; SomnoFlo) and perfused transcardially with 10 mL ice cold dissection buffer (2.5 mM KCl, 0.5 mM CaCl2 dihydrate, 7 mM MgCl2 hexahydrate, 25 mM NaHCO3, 1.25 mM NaH2PO4, 11.6 mM Sodium L-ascorbate, 3.1 mM sodium pyruvate, 110 mM choline chloride, 25 mM glucose). Brains were sectioned at 400 μm on a Vibratome (Leica VT1200 S) and placed in the Brain Slice Keeper (AutoMate Scientific BSK-4) in artificial cerebrospinal fluid (aCSF) (2.5 mM KCl, 118 mM NaCl, 1.3 mM MgCl2, 2.5 mM CaCl2, 26 mM NaHCO3, 1 mM NaH2PO4, 10 mM glucose) constantly perfused with 95% O2 / 5% CO2 gas (carbogen). Slices were allowed to recover at 32°C for 1 hour. For all electrophysiological recordings, slices were allowed to recover for an additional 30 minutes at room temperature in carbogenated aCSF. For all CLIP and RiboTag experiments, slices recovered for an additional 3 hours at room temperature in carbogenated aCSF.

### Electrophysiological Recordings

Glass capillary pipettes were pulled and filled with internal solution for current clamp (130 mM K-Gluconate, 5 mM KCl, 10 mM HEPES, 2.5 mM MgCl2. 4 mM Na2ATP, 0.4 Na3GTP, 10 mM Na-phosphocreatine, 0.6 mM EGTA) or voltage clamp (115 mM CsMeSO3, 20 mM CsCl, 10 mM HEPES, 2.5 mM MgCl2, 4 mM Na2-ATP, 0.4 mM Na-GTP, 10 mM Na-phosphocreatine, and 0.6 mM EGTA). After a giga-ohm seal was achieved, the membrane was disrupted with short bursts of negative pressure to achieve a whole-cell configuration. Rheobase current, the minimal current to elicit an action potential, was determined via stepwise injection of current in 25 pA increments. In voltage clamp recordings, 470 nm LED pulses of 5 ms were delivered to activate ChR2 expressing neurons and to measure LED-induced postsynaptic currents. The minimal LED % illumination required to generate consistent action potential firing and postsynaptic currents varied between 5% - 15%. Recordings were made using a Scientifica SliceScope Pro 1000 with data filtered at 2.4 kHz and digitized at 10 kHz using a Digidata 1440A interface (Molecular Device) driven by pClamp 9.2 (Molecular Devices).

### Immunofluorescence

Mice were deeply anesthetized with 3% inhaled isoflurane (Kent Scientific; SomnoFlo) and perfused transcardially with 10 mL chilled phosphate buffered saline (PBS) followed by 10 mL chilled 4% paraformaldehyde (PFA) in PBS. Brains were then dissected and postfixed in 4% PFA in PBS at 4°C for 24 hours. Brains were then submerged in 30% sucrose for at least 24 hours prior to sectioning. Brains were sectioned using a Leica SM2010R Sliding Microtome at 60 μm. Sections were stored in cryoprotectant solution at −20°C, rinsed thoroughly in PBS and blocked in 5% donkey serum diluted in PBST (1X PBS with 0.3% TritonX-100) for 30 minutes at room temperature. Sections were then incubated in the primary antibody, diluted in PBST at 4°C: Guinea pig anti-NeuN (Millipore ABN90P, 1:1000) and Rabbit anti-HA (Cell Signaling, C29F4, 1:4000). ChR2-mCherry was visualized endogenously without antibody staining. Sections were rinsed with 1X PBS, mounted onto glass slides, dried, and cover slipped with Prolong Gold Antifade. Slides were imaged on a Keyence BZ-X710 fluorescence microscope.

### Ex vivo Optogenetic Activation

Acute brain slices were optogenetically activated using a custom built LED illumination system. This system consisted of a Ultra High Power LED (Prizmatix UHP-T-455-MP) with special 1-inch collimating optics for Microplate Illumination that could be remotely controlled with the benchtop UHP-T-LED Current Controller (Prizmatix) and the Pulser Plus (Prizmatix) to create trains of TTL pulses. The LED height was optimized for homogeneous light distribution to each well. All optogenetic stimulation described in the study consisted of 5 trains of blue light pulses (450–465 nm, peak at 455 nm) at 5 Hz for 30 seconds. Slices were allowed to recover in carbogenated aCSF for 30 minutes after optogenetic stimulation. For Opto-CLIP, slices were then crosslinked on ice, the CA1 hippocampus was rapidly dissected on ice, and tissue was processed for CLIP. For Opto-RiboTag, slices were transferred to cold HBSS + 0.1 mg/mL cycloheximide, the CA1 hippocampus was rapidly dissected on ice, and tissue was processed for RiboTag.

### FMRP-cTag CLIP

Each independent biological replicate (n=4) consisted of hippocampi pooled from 5 Camk2a-Cre;Fmr1-cTag mice aged 11 weeks. Crosslinked tissue was resuspended in 1.5 mL lysis buffer (1X PBS, 1% NP-40, 0.5% NaDOC and 0.1% SDS with cOmplete protease inhibitors (Roche)). Material was homogenized by mechanical homogenization and frozen at 80°C to ensure full cell lysis. Lysates were thawed and subject to DNase treatment (67.5 μl of RQ1 DNase (Promega) per 1.5 mL; 5 minutes; 37°C; 1100 rpm in thermomixer) and RNase treatment (final dilution of 1:1,666,666 of RNase A (Affymetrix); 5 minutes; 37°C; 1100 rpm in thermomixer). Lysate was then clarified by centrifugation at 20,000 × g for 20 min. The resulting supernatant was pre-cleared by rotating with 50 μl Protein G Dynabeads (Invitrogen) (washed in lysis buffer) for 45 min at 4°C. The pre-cleared supernatant was used for immunoprecipitation with 200 μl of Protein G Dynabeads (Invitrogen) loaded with 25 μg each of mouse monoclonal anti-GFP antibodies 19F7 and 19C8^44^. Immunoprecipitation was performed for 2 hr at 4°C with rotation. Beads were then washed twice with lysis buffer, twice with high salt lysis buffer (5X PBS, 1% NP-40, 0.5% NaDOC and 0.1% SDS), twice with stringent wash buffer (15 mM Tris pH 7.5, 5 mM EDTA, 2.5 mM EGTA, 1% TritonX-100, 1% NaDOC, 0.1% SDS, 120 mM NaCl, 25 mM KCl), twice with high salt wash buffer (15 mM Tris pH 7.5, 5 mM EDTA, 2.5 mM EGTA, 1% TritonX-100, 1% NaDOC, 0.1% SDS, 1M NaCl), twice with low salt wash buffer (15 mM Tris pH 7.5, 5 mM EDTA), and twice with PNK wash buffer (50 mM Tris pH 7.4, 10 mM MgCl2, 0.5% NP-40). RNA tags were dephosphorylated with Alkaline Phosphatase (Roche) and subjected to overnight 3’ ligation at 16°C with a pre-adenylated linker (preA-L32)^43^ with the following ligation reaction: 2 μl of 25 mM linker, 2 μl of Truncated T4 RNA Ligase (NEB), 1X ligation buffer (NEB), 2 μl RNase inhibitor (Invitrogen), and 8 μl PEG8000 (NEB). The beads were washed three times with PNK wash buffer and the RNA-protein complexes labeled with 32P with the following reaction: 4 μl 10X PNK buffer (NEB), 2 μl T4 PNK enzyme, 1 μl 32P-γ-ATP at 37°C for 20 minutes shaking at 1100 rpm in a Thermomixer. Beads were then washed three times in PNK/EGTA wash buffer (50 mM Tris pH 7.4, 20 mM EGTA, 0.5% NP-40) and incubated at 70°C for 10 minutes shaking at 1100 rpm in a Thermomixer to eluate RNA/protein complexes from beads and subjected to SDS-PAGE and transfer as described^51^. Regions corresponding to 120–175 kDa were excised from the nitrocellulose membrane and RNA tags were collected with proteinase K/Urea followed by chloroform:isoamyl alcohol (24:1) treatment as previously described^40^. Cloning was performed using the BrdU-CLIP protocol as described^20,21^ using RT primers with six nucleotide barcode index sequences to allow for up to 24 samples to be pooled together in one MiSeq sequencing run (Illumina) and subsequently demultiplexed.

### TRAP- and RNA-seq of acute brain slices

Each independent biological replicate (n=2) consisted of hippocampi pooled from Camk2a-Cre; Rpl22-HA mice aged 11 weeks. Dissected hippocampi from acute brain slices were pooled, resuspended in 5% w/v ice-cold polysome buffer (20 mM HEPES, pH 7.4, 150 mM NaCl, 5 mM MgCl2, 0.5 mM DTT) freshly supplemented with 40 U/ml RNasin Plus (Promega), cOmplete protease inhibitors (Roche), 0.1 mg/mL cycloheximide, and 0.5 mM DTT) and homogenized by dounce homogenization (40 strokes type A pestle followed by 40 strokes type B pestle). NP-40 was added to 1% final concentration and lysate was incubated on ice for 10 min. Supernatant was subsequently centrifuged at 2000 × g for 10 min at 4°C followed by 20,000 × g for 10 min at 4°C. 10% of the resulting lysate was used as input and the remaining lysate was pre-cleared by rotation with 50 μl Protein G Dynabeads (Invitrogen) (washed in polysome buffer) for 45 min at 4°C. HA-tagged ribosomes were collected by indirect IP by adding mouse anti-HA (8 μl antibody per mL lysate; Biolegend; MMS-101R) antibody directly to the lysate, rotating the mixture at 4°C for 4 hours, and adding 150 μl Protein G dynabeads to the lysate + antibody followed by overnight rotation at 4°C. Beads were washed twice with polysome buffer containing 1% NP-40 and three times with high salt wash buffer (20 mM HEPES, 5 mM MgCl2, 350 mM KCl, 1% NP-40) freshly supplemented with 0.1 mg/mL cycloheximide, and 1 mM DTT. All washes were 5 minutes with rotation at 4°C. RNA was extracted from input and IP samples by incubation with 1 mL Trizol at room temperature for 5 minutes and then RNeasy Lipid Tissue Mini Kit (Qiagen) via the manufacturer’s protocol with on-column DNase treatment (Roche). RNA quantity and quality were obtained by the Agilent 2100 Pico Bioanalyzer system. The libraries were prepared by Illumina Stranded Total RNA Prep with Ribo-Zero Plus following the manufacturer’s instructions. High-throughput sequencing was performed on NovaSeq (Illumina) to obtain 150 nucleotide paired-end reads.

### Bioinformatics

#### Opto-RiboTag

Transcript expression was quantified Input and IP samples using salmon^52^ and mm10 gene models. Differential expression analysis was performed using DESeq2^53^. Transcripts were considered significantly enriched (IP vs input; activated vs control) with a Benjamini–Hochberg FDR less than 0.05.

#### Opto-FMRP-CLIP

CLIP reads were processed as described previously using CLIP Tool Kit (CTK)^51,54^. Briefly, raw reads were filtered for quality and demultiplexed using indexes introduced during the reverse transcription reaction. PCR duplicates were collapsed and adapter sequences removed. Reads were mapped to the mm10 RefSeq genome using BWA^55^. Mapped reads were further collapsed using fastq2collapse.pl from the CTK toolkit with default options. The distribution of CLIP reads that uniquely map to the genome (Figure 4G) was determined using bed2annotation.pl from the CTK toolkit.

#### CLIP normalization using RiboTag

RiboTag transcript per million (TPM) was determined for each transcript using salmon^52^ and a single transcript with the highest mean TPM across all activated and control replicates was selected per gene. This set of 14729 transcripts was used for all subsequent analysis. CLIP tags were preprocessed as described above and then were mapped to the transcriptome using STAR^56^ with --quantMode GeneCounts TranscriptomeSAM. Mapped reads were collapsed using fastq2collapse.pl from the CTK toolkit with default options. Only reads that mapped to transcripts expressed by RiboTag were retained. CLIP scores were calculated as described previously^20,21^. Briefly, for each replicate, CLIP TPM was calculated based on transcript length and library size. Scatter plots of log2 CLIP TPM vs log2 RiboTag TPM for each condition and replicate were made and a linear regression line fitted to the data. The CLIP score for each transcript and each replicate was then calculated from the slope (lm slope) and intercept (lm intercept) of the fitted line using the equation: CLIP score= log2 CLIP TPM - (lm slope × RiboTag TPM) + lm intercept. The final CLIP score for each transcript was determined as the mean CLIP score across all biological replicates. Transcripts were classed as stringent targets if they had a CLIP score 2 across all biological replicates, high binding targets if they had a CLIP score greater than 1 but less than 2, low binding targets if they had a CLIP score between 0 and 1. All other transcripts were classed as non-targets. Differential comparison of CLIP scores between resting and activated conditions was done with limma^57^.

#### GO and GSEA analysis

GeneGene ontology (GO) analysis was performed using the function enrichGO in the R package clusterProfiler^58^. GSEA analysis was done using the function gseGO in the R package clusterProfiler^58^. The input for GSEA was log2 fold change of CLIP scores in activated versus resting conditions determined by limma^57^.

#### Code availability

All customized R scripts have been deposited on the public Github repository: https://github.com/ruthasinger

## Supplementary Material

Supplement 1

## Figures and Tables

**Key Resources Table:** 

Reagent or Resource	Source	Identifier	Additional Details
*Antibodies*			
Guinea pig anti-NeuN	Millipore (ABN90P)	AB_2341095	IF (1:2000)
Mouse anti-HA	Biolegend (MMS-101R)	AB_291262	IP (8 μl/mL lysate)
Rabbit anti-HA tag	Cell Signaling Technology (3724)	AB_1549585	IF (1:1000)
Mouse anti-GFP, HtzGFP19F7	Heiman et al 2008	AB_2716736	IP (25 μg/mL lysate)
Mouse anti-GFP, HtzGFP19C8	Heiman et al 2008	AB_2716737	IP (25 μg/mL lysate)
Mouse anti-BrdU	Abcam (ab8955)	AB_306886	CLIP (5 μg/RT rxn)
*Virus Strains*			
pAAV-EF1a-DIO-hChR2-mCherry-WPRE-HGHpA	Addgene (Karl Deisseroth)	Addgene_20297	1:20 dilution
pAAV-FLEX-tdTomato	Addgene (Edward Boyden)	Addgene_28306	1:20 dilution
*Critical Commercial Assays*			
RNeasy Lipid Tissue Mini Kit	Qiagen	74804	
Agilent RNA 6000 Pico Kit	Agilent	5067-1513	
Illumina Stranded Total RNA Prep with Ribo-Zero Plus	Illumina	20040525	
*Deposited Data*			
FMRP-CLIP and RiboTag sequencing data from CA1 cell bodies and neurities	Hale et al 2021^21^	GEO: GSE174303	
*Experimental models: Organisms/strains*			
B6.Cg-Tg(Camk2a-cre)T29-1Stl/J	Jackson Laboratory	IMSR_JAX:005359	
B6N.129-Rpl22tm1.1	Jackson Laboratory	IMSR_JAX:011029	
Fmr1-cTag	Van Driesche et al. 2019^37^		
*Software and algorithms*			
Pulser Plus	Prizmatix		
pClamp	Molecular Devices	SCR_011323	
Salmon	Patro et al 2017^52^	SCR_017036	
DESeq2	Love et al 2014^53^	SCR_015687	
CLIP Tool Kit (CTK)	Shah et al 2014^54^	SCR_019034	
BWA	Li et al 2009^55^	SCR_010910	
STAR	Dobin et al 2013^56^	SCR_004463	
Limma	Ritchie et al 2015^57^	SCR_010943	
ClusterProfiler	Yu et al 2012^58^	SCR_016884	
