# Supplementary material for: Opto-CLIP reveals dynamic FMRP regulation of mRNAs upon CA1 neuronal activation
Source: bioRxiv. 2025 Jan 15:2024.08.13.607210. Originally published 2024 Aug 14. Preprint. [Version 2] doi: 10.1101/2024.08.13.607210 (PMC11343148; doi:10.1101/2024.08.13.607210)
Supplement: Supplement 1 [file NIHPP2024.08.13.607210v2-supplement-1.pdf]

bioRxiv preprint doi: https://doi.org/10.1101/2024.08.13.607210; this version posted January 15, 2025. The copyright holder for this preprint (which was not certified by peer review) is the author/funder, who has granted bioRxiv a license to display the preprint in perpetuity. It is made available under aCC-BY 4.0 International license.

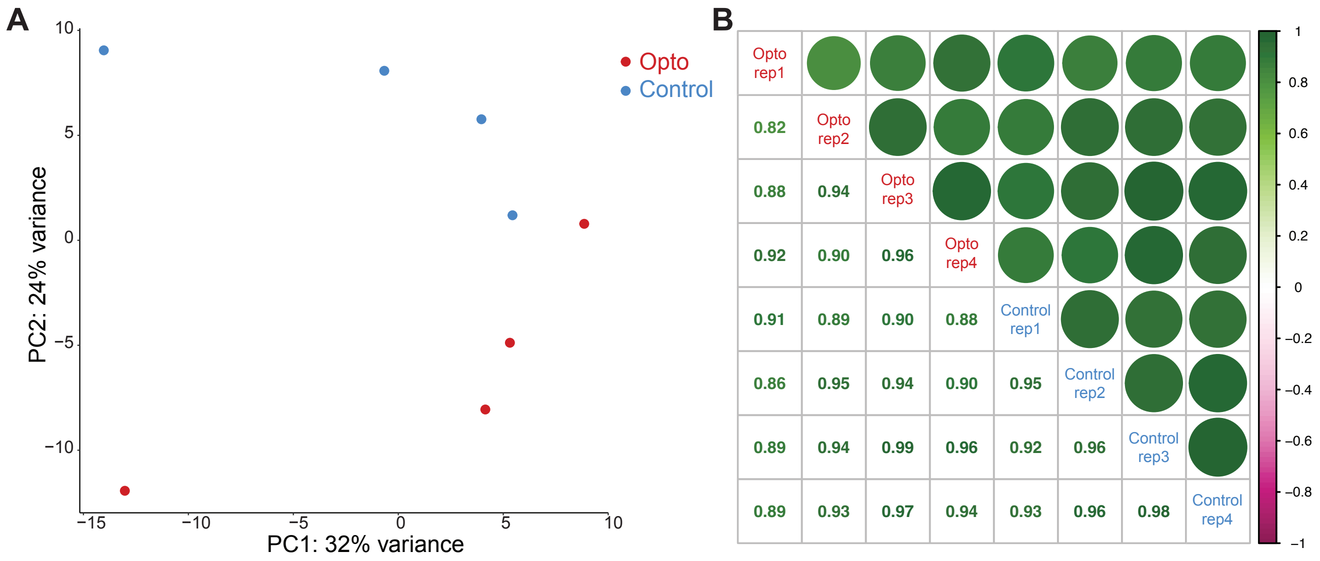

Supplemental Figure 1. A) Principal component analysis (PCA) shown for Opto-FMRP-CLIP samples (red) and Control FMRP-CLIP samples (blue). B) Correlation plots of each CLIP sample compared to each other. Color of the circle corresponds to the r-squared value, which is listed in the matching box on the other half of the matrix. Opto samples are listed in red text and Control samples are in blue text (n=4).

bioRxiv preprint doi: https://doi.org/10.1101/2024.08.13.607210; this version posted January 15, 2025. The copyright holder for this preprint (which was not certified by peer review) is the author/funder, who has granted bioRxiv a license to display the preprint in perpetuity. It is made available under aCC-BY 4.0 International license.

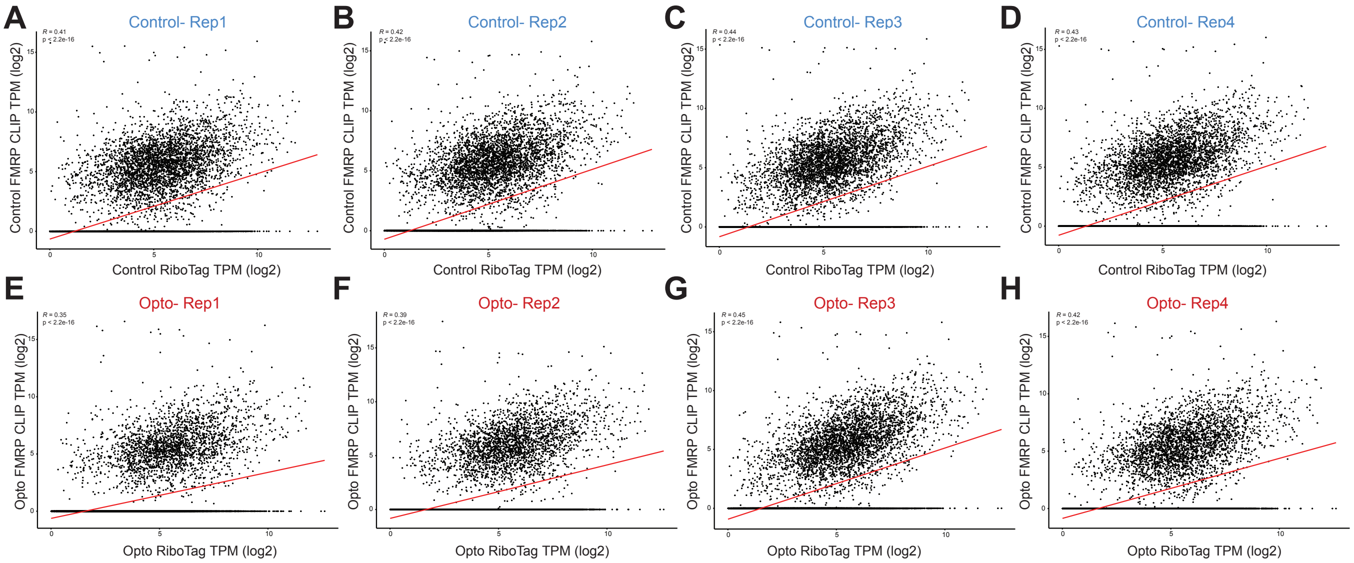

Supplemental Figure 2. A and B) FMRP-CLIP targets were defined by normalizing CLIP tag density across the coding region to the relative abundance of the transcript as measured by RiboTag. A CLIP score per transcript was calculated independently for each of the eight CLIP biological replicates. Scatter plots of $\log_2$ normalized transcript per million (TPM) of CLIP tags from an individual Control (A) or Opto-FMRP-CLIP (B) experiment compared to average $\log_2$ RiboTag TPM from Control (A) or Opto-RiboTag (B). n=4 biological replicates for FMRP-CLIP. n=3 biological replicates for RiboTag. Red line indicates the linear regression model used to calculate CLIP scores. See methods for further details on CLIP score calculations.

bioRxiv preprint doi: https://doi.org/10.1101/2024.08.13.607210; this version posted January 15, 2025. The copyright holder for this preprint (which was not certified by peer review) is the author/funder, who has granted bioRxiv a license to display the preprint in perpetuity. It is made available under aCC-BY 4.0 International license.

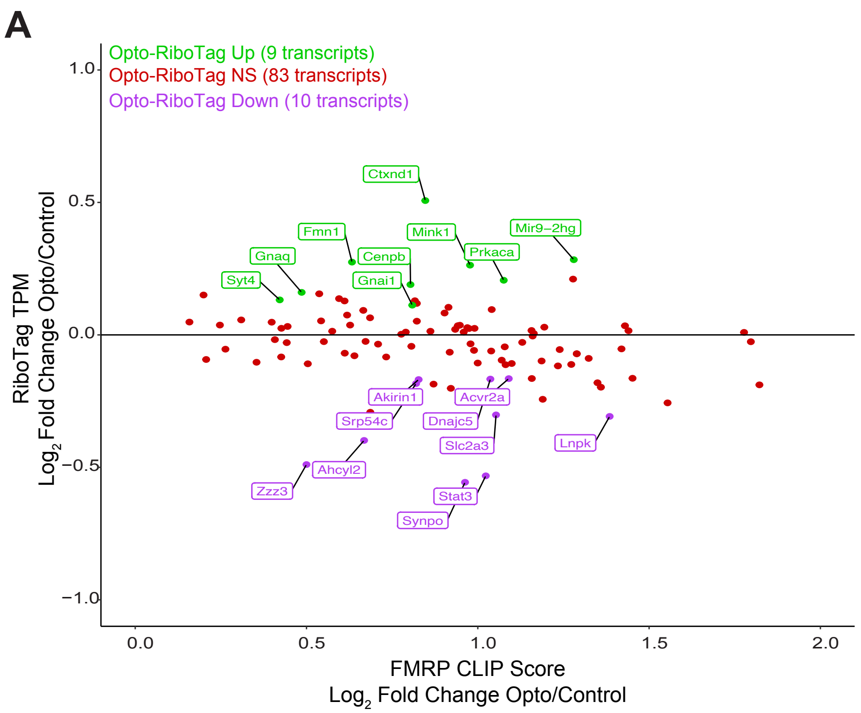

Supplemental Figure 3. A) Scatter plot of Opto-RiboTag versus Control RiboTag ($\log_2$ TPM) compared to Opto-CLIP versus Control CLIP CLIP scores ($\log_2$ fold change). Red dots indicate transcripts that are more bound by FMRP in activated neurons (FMRP-Up) and are unchanged by Opto RiboTag. Green dots indicate transcripts that are more bound by FMRP in activated neurons (FMRP-Up) and are upregulated in activated RiboTag. Purple dots indicate transcripts that are more bound by FMRP in activated neurons (FMRP-Up) and are downregulated in activated RiboTag.

bioRxiv preprint doi: https://doi.org/10.1101/2024.08.13.607210; this version posted January 15, 2025. The copyright holder for this preprint (which was not certified by peer review) is the author/funder, who has granted bioRxiv a license to display the preprint in perpetuity. It is made available under aCC-BY 4.0 International license.

Reagent or Resource	Source	Identifier	Additional Details
Antibodies			
Guinea pig anti-NeuN	Millipore (ABN90P)	AB_2341095	IF (1:2000)
Mouse anti-HA	Biolegend (MMS-101R)	AB_291262	IP (8 µl/mL lysate)
Rabbit anti-HA tag	Cell Signaling Technology (3724)	AB_1549585	IF (1:1000)
Mouse anti-GFP, HtzGFP19F7	Heiman et al 2008	AB_2716736	IP (25 µg/mL lysate)
Mouse anti-GFP, HtzGFP19C8	Heiman et al 2008	AB_2716737	IP (25 µg/mL lysate)
Mouse anti-BrdU	Abcam (ab8955)	AB_306886	CLIP (5 µg/RT rxn)
Virus Strains			
pAAV-EF1a-DIO-hChR2-mCherry-WPRE-HGHpA	Addgene (Karl Deisseroth)	Addgene_20297	1:20 dilution; 1.25 E+09 GC per site
pAAV-FLEX-tdTomato	Addgene (Edward Boyden)	Addgene_28306	1:20 dilution; 1.25 E+09 GC per site
Critical Commercial Assays			
RNeasy Lipid Tissue Mini Kit	Qiagen	74804	
Agilent RNA 6000 Pico Kit	Agilent	5067-1513	
Illumina Stranded Total RNA Prep with Ribo-Zero Plus	Illumina	20040525	
Deposited Data			
FMRP-CLIP and RiboTag sequencing data from CA1 cell bodies and neurities	Hale et al 2021	GEO: GSE174303	
Opto-FMRP-CLIP	This study	GEO: GSE286379	
Opto-RiboTag	This study	GEO: GSE286381	
Experimental models: Organisms/strains			
B6.Cg-Tg(Camk2a-cre)T29-1Stl/J	Jackson Laboratory	IMSR_JAX:005359	
B6N.129-Rpl22tm1.1	Jackson Laboratory	IMSR_JAX:011029	
Fmr1-cTag	Van Driesche et al. 2019		
Software and algorithms			
Pulser Plus	Prizmatix		
pClamp	Molecular Devices	SCR_011323	
Salmon	Patro et al 2017	SCR_017036	
DESeq2	Love et al 2014	SCR_015687	
CLIP Tool Kit (CTK)	Shah et al 2014	SCR_019034	
BWA	Li et al 2009	SCR_010910	
STAR	Dobin et al 2013	SCR_004463	
Limma	Ritchie et al 2015	SCR_010943	
ClusterProfiler	Yu et al 2012	SCR_016884	

Supplemental Table 1. Key resources used in this study.
