# Supplementary material for: Opto-CLIP reveals dynamic FMRP regulation of mRNAs upon CA1 neuronal activation
Source: bioRxiv. 2024 Aug 14:2024.08.13.607210. Preprint. [Version 1] doi: 10.1101/2024.08.13.607210 (PMC11343148; doi:10.1101/2024.08.13.607210)
Supplement: Supplementary file 1 [file NIHPP2024.08.13.607210v1-supplement-1.pdf]

bioRxiv preprint doi: https://doi.org/10.1101/2024.08.13.607210; this version posted August 14, 2024. The copyright holder for this preprint (which was not certified by peer review) is the author/funder, who has granted bioRxiv a license to display the preprint in perpetuity. It is made available under aCC-BY 4.0 International license.

SUPPLEMENTARY FIGURES

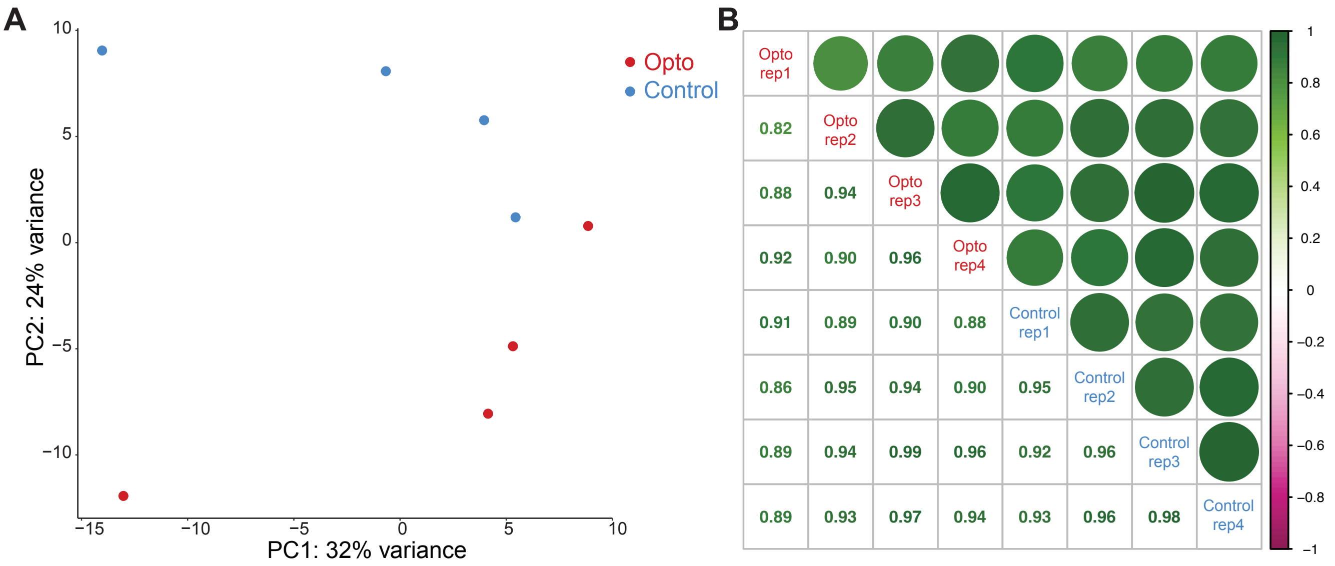

Figure S1
A) Principal component analysis (PCA) shown for Opto-FMRP-CLIP samples (red) and Control FMRP-CLIP samples (blue).
B) Correlation plots of each CLIP sample compared to each other. Color of the circle corresponds to the r-squared value, which is listed in the matching box on the other half of the matrix. Opto samples are listed in red text and Control samples are in blue text (n=4).

bioRxiv preprint doi: https://doi.org/10.1101/2024.08.13.607210; this version posted August 14, 2024. The copyright holder for this preprint (which was not certified by peer review) is the author/funder, who has granted bioRxiv a license to display the preprint in perpetuity. It is made available under aCC-BY 4.0 International license.

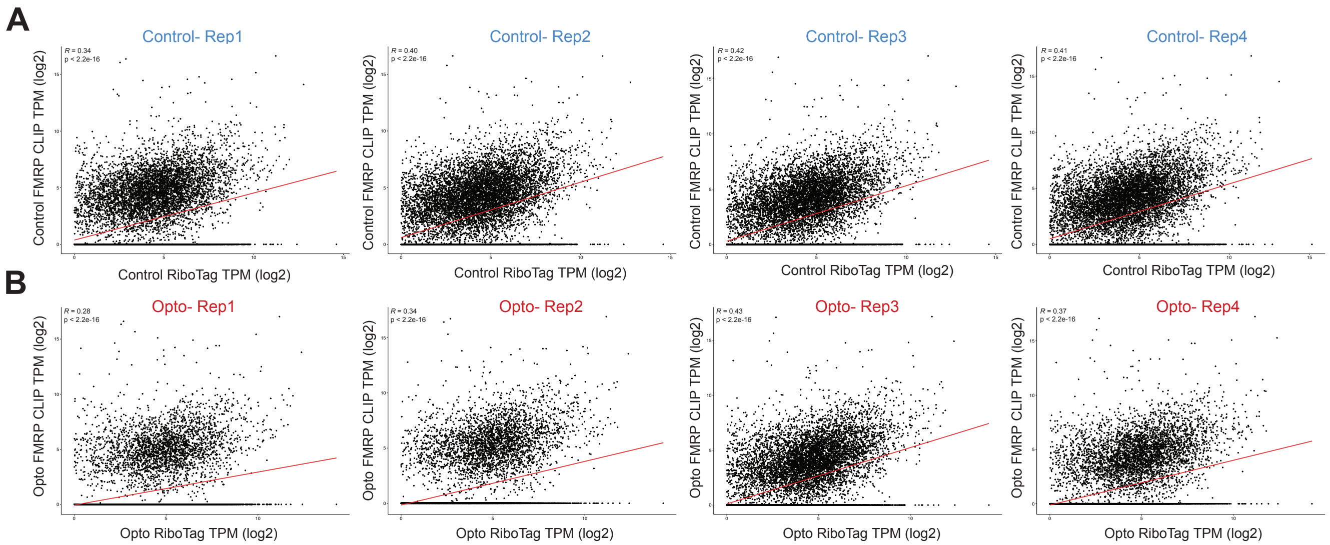

Figure S2.
A and B) FMRP-CLIP targets were defined by normalizing CLIP tag density across the coding region to the relative abundance of the transcript as measured by RiboTag. A CLIP score per transcript was calculated independently for each of the eight CLIP biological replicates. Scatter plots of $\log_2$ normalized transcript per million (TPM) of CLIP tags from an individual Control (A) or Opto-FMRP-CLIP (B) experiment compared to average $\log_2$ RiboTag TPM from Control (A) or Opto-RiboTag (B). n=4 biological replicates for FMRP-CLIP. n=2 biological replicates for RiboTag. Red line indicates the linear regression model used to calculate CLIP scores. See methods for further details on CLIP score calculations.
